# Effect of long-term use of antipsychotics on the ventricular repolarization index

**DOI:** 10.1186/s12888-024-05947-1

**Published:** 2024-07-16

**Authors:** Qiong Liu, Xiaoping Yuan, Chengdong Sheng, Weixun Cai, Xuhong Geng, Huanzhong Liu, Suqi Song

**Affiliations:** 1 Department of ECG Room, Chaohu Hospital of Anhui Medical University, 64 North Chaohu Road, Hefei, 238000 China; 2 Department of Psychiatry, Chaohu Hospital of Anhui Medical University, 64 North Chaohu Road, Hefei, 238000 China; 3 Anhui Psychiatric Center, Anhui Medical University, Hefei, 238000 China; 4 Heart Center, Department of Electrocardiographic & Cardiac Examination, Zhejiang Provincial People’s Hospital(Affiliated People’s Hospital), Hangzhou Medical College, Hangzhou, 310000 Zhejiang China; 5 Department of Function, Fourth Hospital of Hebei Medical University, Shijiazhuang, 050011 China

**Keywords:** Antipsychotics, Ventricular repolarization index, Arrhythmia, Sudden cardiac death

## Abstract

**Background:**

The risk of arrhythmia is usually assessed by the length of the corrected QT interval (QTc) when patients use antipsychotics. Prolonged QTc intervals are thought to increase the probability of malignant ventricular arrhythmias, and if we focus only on the QTc interval, we may be influenced by a single factor and make decisions that are not conducive to effective treatment. The index of cardiac electrophysiological balance (iCEB) is considered more valuable than the QTc for predicting drug-induced arrhythmias. It has been used in clinical practice, but no studies have observed changes in this index after the use of antipsychotics.

**Objective:**

To investigate the changes in ventricular repolarization indices and the occurrence of arrhythmias in patients who have been using antipsychotic drugs for a long time, to compare the changes in iCEBc and QTc and to predict abnormal iCEBc values.

**Methods:**

Patients with schizophrenia who had been hospitalized for more than 4 years and who were receiving atypical antipsychotics underwent a 12-lead synchronized electrocardiogram (ECG) every 2–4 weeks. The baseline data were measured at admission, defined as the baseline (time0), and the most obvious abnormal changes in ventricular depolarization and repolarization measured every 12 months were one-year follow-up (time1), two-year follow-up (time2), three-year follow-up (time3), and four-year follow-up (time4). Repeated measures analysis of variance was used for comparisons. The types and doses of drugs taken at 5 time points were recorded and converted into chlorpromazine equivalents for comparison. The incidence of arrhythmia during the observation cycle was recorded.

**Results:**

The patients had been treated with antipsychotic medication for 4 years, and the duration of the QRS wave was longer in males than in females. TpTe, TpTe/QRS, TpTe/QT, TpTe/QTc, iCEB, and iCEBc increased significantly with hospital stay, while TpTe, TpTe/QRS, TpTe/QT, and TpTe/QTc exhibited more obvious changes in these indicators in female patients (*P* < 0.01). The changes in iCEB and iCEBc were more significant in males (*P* < 0.01). The incidences of arrhythmia (arrhythmic events included premature ventricular beats and premature atrial beats) within 5 time points were 2.5%, 6.25%, 6.25%, 6.25% and 5%, respectively. More than 90% of patients treated with antipsychotics did not have any arrhythmias. No TdP syncope or other cardiovascular symptoms were found in any of the patients.

**Conclusion:**

After long-term use of antipsychotics, the ventricular repolarization index gradually increased with time. The new ventricular repolarization indices iCEB and iCEBc were more sensitive than the QTc at predicting arrhythmia. According to the abnormal QTc values in men and women, we predict that the abnormal value of the iCEBc in males is 4.528 and that in females is 5.315.

**Supplementary Information:**

The online version contains supplementary material available at 10.1186/s12888-024-05947-1.

## Introduction

Relative to that of the general population, the average life expectancy of people with schizophrenia decreases by 15–20 years. A national retrospective cohort study conducted in Denmark showed that psychiatric patients have four times the risk of sudden cardiac death of other populations, nearly half of whom develop heart disease before death [1]. In 2009, Ray et al. published a retrospective cohort study based on large samples of first- and second-generation antipsychotics and reported that patients aged between 30 and 74 years who were treated with antipsychotics had a greater risk of sudden cardiac death [2]. Similarly, Chen et al. showed that antipsychotic polypharmacy did not contribute to increased mortality in patients with a psychotic disorder compared to patients without antipsychotic medication, but antipsychotic polypharmacy may still be associated with increased mortality compared to that of the general population [3]. Therefore, it is necessary to evaluate the safety of antipsychotic drugs.

The use of antipsychotics can prolong ventricular repolarization. Prolonged ventricular repolarization can induce malignant arrhythmias, such as apical torsion de pointes (TdP) and sudden cardiac death [4–6]. Antipsychotics can prolong ventricular repolarization by inhibiting the ion channel of the ventricular myocyte membrane, and prolongation of the QT interval can be observed by ECG [7]. The Chinese guidelines for the prevention and treatment of schizophrenia define QTC ≥ 450 ms in adult males, ≥ 470 ms in adult females, or elevated 60 ms compared to the baseline as a risk event, and the patient must stop taking drugs or switch to drugs that have little effect on QTc [8]. However, if we only evaluate QT intervals, we may abandon some effective treatments and may also overlook some important information. The QTc interval reflects the global depolarization repolarization time of the ventricle and does not reflect the local electrophysiological heterogeneity of the ventricle [9, 10]. The Bazett formula is also commonly used to calculate QTc (QTc = QT/√RR). When the ventricular rate is fast, the QTc value often exceeds the actual value, which may lead to the occurrence of a “false positive”. When the ventricular rate increases to a certain extent, the limitations of the way QTc is calculated bias the results, and QTc loses its predictive value for TdP risk [11, 12]. The TpTe interval is the interval between the apex of the T-wave and the terminal point of the T-wave on the surface ECG, reflecting local changes in ventricular muscle action potentials. Local action potential prolongation may be more relevant to the development of arrhythmias than an increase in overall repolarization time. Overall and local repolarization are often affected by the same factors (e.g., myocardial ischaemia), and ectopic beats often come from the region with the greatest heterogeneity in local repolarization; however, QTc cannot distinguish between overall and local repolarization changes. Heart rate correction, the ECG measurement method and the selection of ECG leads are the main factors influencing the accuracy of QTc. Heart rate correction did not affect the association of the TpTe interval with mortality, all-cause deaths had longer peak-propensity intervals than did survival, and patients with longer TpTe had a greater risk of all-cause death than did those with shorter TpTe [13].

The index of cardiac electrophysiological balance (iCEB), a predictive marker for drug-induced cardiac arrhythmia, is used to determine the balance between repolarization and depolarization of the ventricular muscle. It is the product of the conduction velocity of excitation in the ventricular muscle and the effective refractory period of the ventricular muscle. It can be simply calculated as QT/QRS [14]. The QRS wave group reflects the depolarization process of the left and right ventricles, the QT interval reflects the total duration of ventricular depolarization and repolarization, and the iCEB reflects the balance of depolarization and repolarization. The incidence of iCEB often increases in patients with long QT syndrome (LQTS), and those with Brugada syndrome have a lower incidence of iCEB than those with negative gene mutations in the same family. Like the corrected QT interval (QTc), the iCEB can also be corrected by the heart rate (iCEBc). It is a marker for assessing the risk of arrhythmia [15, 16].

It has been reported that sex hormones have different effects on ventricular repolarization indices. Moreover, testosterone in men and progesterone in women can shorten the duration of ventricular action potentials [17, 18]. Few studies have investigated these phenomena in schizophrenia patients, so we designed this study to evaluate the sensitivity of the new ventricular repolarization index for predicting arrhythmia, as well as the different changes in the ventricular repolarization index between men and women, and to explore the abnormalities in the values of the iCEBc between the two sexes.

## Methods

### Participants

This was a retrospective study that ran from January 2017 to September 2023. All patients were hospitalized in the Department of Psychiatry, Chaohu Hospital Affiliated with Anhui Medical University. This study was approved by the Human Research and Ethics Committee of Chaohu Hospital Affiliated with Anhui Medical University (No. KYXM-202305-010). After a detailed description of our study, informed consent was obtained from the patient and guardian.

The inclusion criteria for patients were as follows: (1) aged 18–65 years, either sex; (2) had a diagnosis of schizophrenia for the first time or as a relapse according to the Diagnostic and Statistical Manual of Mental Disorders (DSM-5) diagnostic criteria, and patients who relapsed were not taking any antipsychotic medication prior to admission to the hospital; (3) had been continuously hospitalized for more than 4 years; and (4) had combined use of atypical antipsychotics during hospitalization.

The exclusion criteria were as follows: (1) had severe mental disorders other than schizophrenia according to the DSM-5 criteria, such as delusional disorders, II bipolar and related disorders, depressive disorders, neurodevelopmental disorders, substance-related and addictive disorders, or personality disorders, etc. (2) had abnormal liver and kidney function, a history of drug allergy, a history of percutaneous coronary intervention or valvular surgery, cardiac dysfunction, hyperthyroidism or hypothyroidism, implantable cardiac devices, severe infection, malignant tumours, or uncorrected electrolyte disorders; (3) had arrhythmia, conduction block, atrial or ventricular hypertrophy, or hereditary ion channel diseases such as LQTS; and (4) were pregnant or lactating.

### Measurement

#### Measurement of ECG data

During hospitalization, these patients were not allowed to consume alcohol, tobacco or caffeinated drinks, and 12-lead ECGs were recorded synchronously every 2–4 weeks. The examination time was between 10:00 and 15:00. The ECG data were examined and recorded on admission, defined as baseline (time 0). Afterwards, the ECG data were collected every 12 months. Since the electrocardiogram was measured every 2–4 weeks, there may be two electrocardiograms in the 12th month. If there were two electrocardiograms, a longer QT/QTC value or one with arrhythmia was selected to measure and analyse the data. Each time point was defined as one year of follow-up (time 1), two years of follow-up (time 2), three years of follow-up (time 3), or four years of follow-up (time 4). The electrocardiogram was recorded by using an American DMS ECG workstation to collect the 12-lead synchronous resting ECG, which was recorded at a chart speed of 25 mm/second for more than 20 s. The voltage was 10 mm/mV, and the collected data were saved to the computer. The ECG parameters were measured manually by two experienced doctors. The measurement data came from leads II and V5. Three stable and clear P-QRS-T wave groups were selected for each lead, and the QRS wave duration, RR interval, QT interval, QTc interval, TpTe interval, TpTe/QTc ratio, TpTe/QT ratio, iCEB and iCEBc were measured or calculated. The average of the three data points was taken as the measurement result.

The space between two adjacent R waves is an RR interval. The time from the QRS wave to the end of the T wave is the QT interval. The intersection of the T wave and the baseline was used to determine the endpoint of the T wave. When the T wave has not returned to the baseline U wave, the T wave ends when the descending extension line intersects at the intersection of the baseline extension line.

**Fig. 1:**
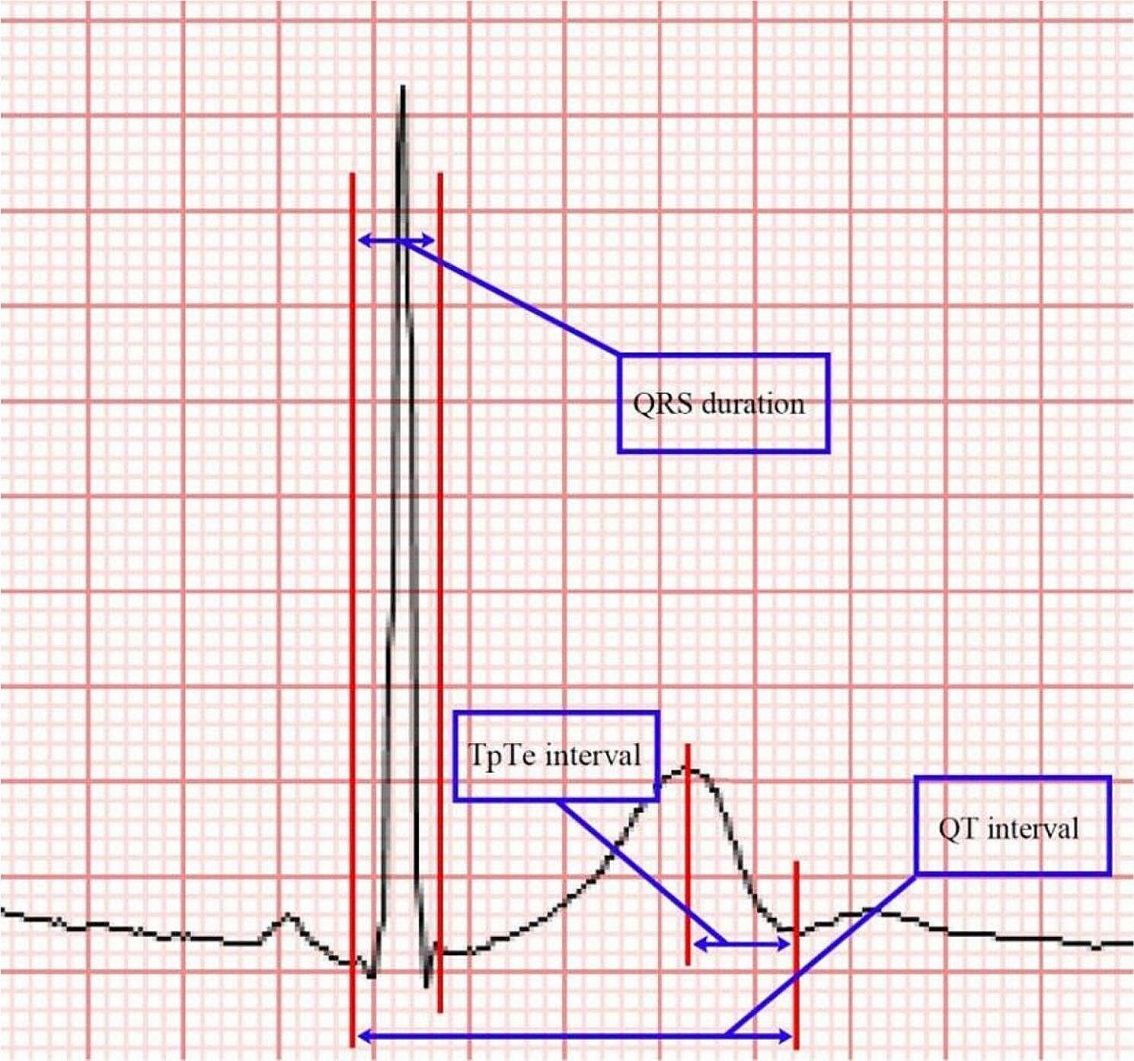
Measurement range of the ECG data

The Bazett formula was used to calculate the QTc (QTc = QT/RR). TpTe is the time between the peak of the T wave and its end. iCEB = QT/QRS, and iCEBc = QTc/QRS. All arrhythmic events shown in the selected ECG were recorded [21] (Fig. 1).

#### Measurement of other data

During the recording of the ECG data, blood samples were taken to detect liver function, renal function, blood parameters, blood lipids, electrolytes and blood concentrations of drugs, and the data were recorded at that time to determine whether there were metabolic problems. The types and doses of atypical antipsychotics were recorded. The types of medications used in these patients included olanzapine, risperidone, aripiprazole, ziprasidone, clozapine, amisulpride, quetiapine fumarate, and paliperidone extended-release tablets, which were converted to chlorpromazine equivalents according to the dose. The drug data for the first year are the maximum drug dose in the first month after admission, which is converted into chlorpromazine equivalent, and then the chlorpromazine equivalent at each time point is converted to the maximum drug dose used in the month with an interval of 12 months from the previous time point.

Changes in blood glucose, blood lipids and blood pressure were recorded. Hypertension was defined as a systolic/diastolic blood pressure ≥ 140/90 mmHg (1 mmHg = 0.133 kPa) and/or a diagnosis of hypertension; type 2 diabetes was defined as a venous blood glucose ≥ 11.1 mmol/L at any time and fasting venous blood glucose ≥ 7.0 mmol/L. Hyperlipidaemia was defined as triglyceride (TG) ≥ 2.26 mmol/L, total cholesterol (TC) ≥ 6.22 mmol/L, high-density lipoprotein cholesterol (HDL-c) < 1.04 mmol/L or low-density lipoprotein cholesterol (LDL-c) ≥ 4.14 mmol/L. Any of the above four abnormalities can be defined as hyperlipidaemia. Serum potassium and calcium levels were assessed to exclude their roles in the development of arrhythmias. At the time of admission, we collected data on the patients’ age, course of schizophrenia, and past alcohol and cigarette use.

### Statistical analysis

Our measurement data are presented as the mean ± standard deviation, and categorical variables are presented as percentages. The patients were divided into two groups according to sex. Student’s t test was used for quantitative variables, and the chi-square test was used for qualitative variables. Age, duration of schizophrenia, alcohol and tobacco use before admission and concomitant diseases after admission were analysed in the two groups. The S‒W test was used to test for normality, and repeated measures analysis of variance (RANOVA) was used to compare the changes in ventricular depolarization, repolarization and chlorpromazine equivalents between the two sexes. The dependent variables were repeated within the effects of ventricular depolarization, repolarization and the chlorpromazine equivalent measured at 5 time points, as well as between effects of the two sexes. A history of smoking before admission was a covariate. According to the repeated measures analysis of variance (ANOVA) model, the independent variables were ventricular depolarization, repolarization index and chlorpromazine equivalent. There were significant differences in the interaction between sex and time, so simple effect analysis was carried out with the index of the baseline value as the covariable to check the difference in the result index at different time points (time 1, time 2, time 3 and time 4). If the sex×time interaction is not significant, no further statistical test is needed. To adjust for multiple tests, Bonferroni correction was applied. In the analysis of variance of repeated measurements, the Greenhouse–Geissler correction was used to adjust the degree of freedom to correct the violation of sphericity. The ventricular repolarization index was screened by receiver operating characteristic (ROC) curve analysis. Statistical calculations were performed with SPSS 26.0, and two-tailed *P* < 0.05 was considered to indicate statistical significance. One-way RANOVA was used to compare the differences in ventricular depolarization and repolarization indices between the two sexes at different time points, and GraphPad Prism version 9.0 was used to construct all the figures.

## Results

### General information of the study participants

**Fig. 2:**
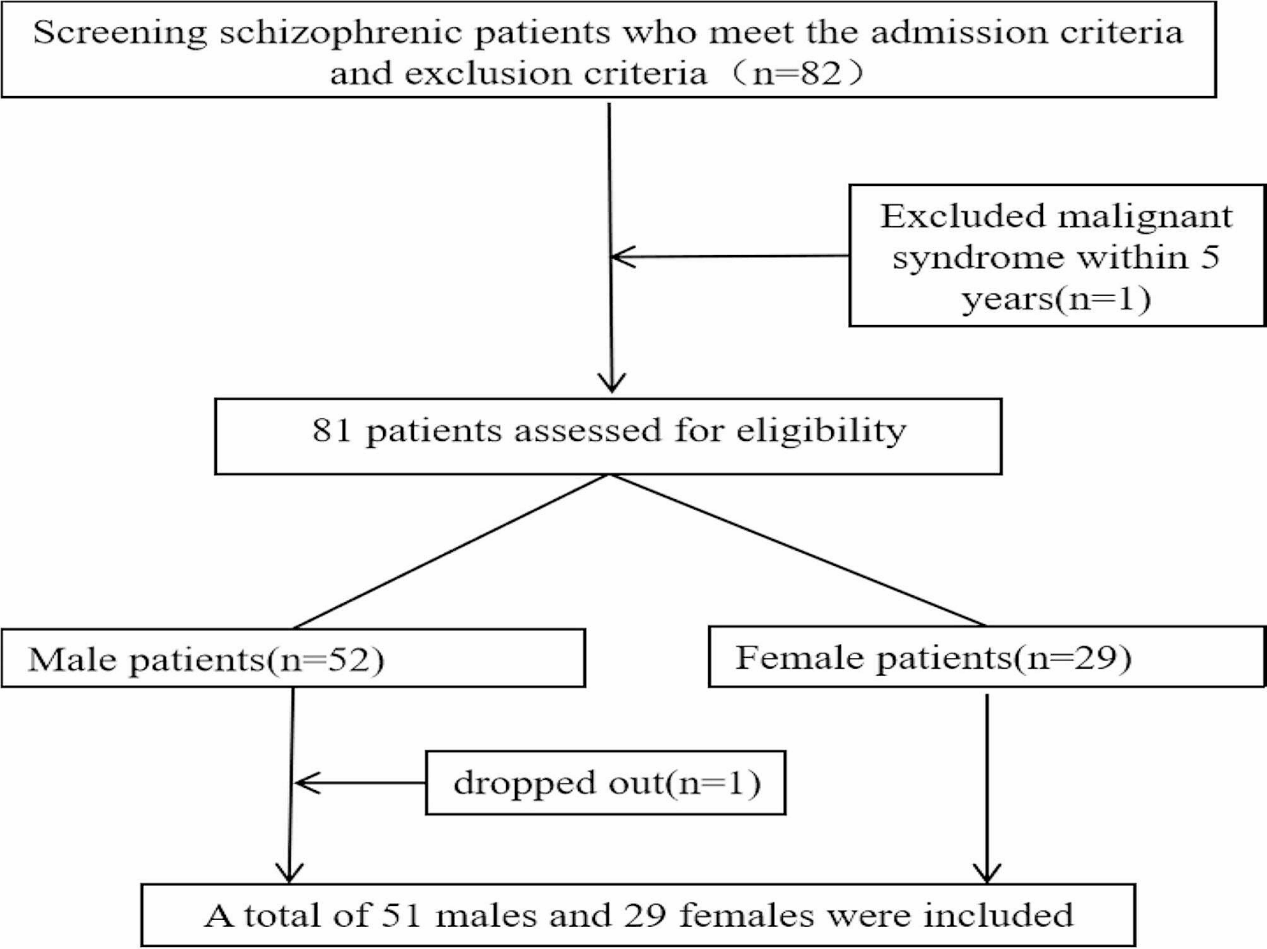
Flow chart of the research

Eighty patients were included in this study, and the specific screening process is shown in Fig. 2. A total of 51 male patients and 29 female patients were included. There was no significant difference in age, history of schizophrenia or alcohol consumption before admission between the sexes. Significantly more males than females used tobacco before admission (*P* < 0.01). As a policy in inpatient wards, patients are prohibited from using tobacco and alcohol. During the more than 4-year study period, these patients lived in the same hospital environment and had a uniform diet distribution and similar activities and lifestyles. There were no significant differences in hypertension, type 2 diabetes or hyperlipidaemia between the two groups (Table 1).

**Table 1:** General information and comorbidities during hospitalization in schizophrenia patients of different sexes **:*p* < 0.01

	Male	Female	t/χ^2^	*P*
Number	51	29		
Age (years), mean ± sd	43.04 ± 10.26	43.36 ± 12.20	-0.12	0.91
Duration of schizophrenia (years), mean ± sd	15.80 ± 8.05	17.86 ± 11.84	-0.82	0.42
Alcohol use(before hospitalization), n(%)	10(19.6)	2(6.9)	2.34	0.13
Tobacco use (before hospitalization), n (%)	24(47.1)	0(0)	19.49	0.00^**^
Comorbid condition, n(%)				
Hypertension	6(11.8)	3(10.3)	0.04	0.85
Type 2 diabetes	8(15.7)	6(20.7)	0.32	0.57
Hyperlipidaemia	12(23.5)	4(13.8)	1.09	0.30

**: *p* < 0.01.

### Comparisons of ECG data and the chlorpromazine equivalent dose between different sexes at different timepoints

The QRS duration was longer in males than in females (*F*_sex_=22.09, *P*_sex_=0.00), but the difference between different timepoints was not significant, nor was the difference in RR intervals between the sexes. The QT, QTc, iCEB, and iCEBc were significantly greater (*P* < 0.05), and there were intergroup differences in iCEB and iCEBc (all *P* < 0.01). There were significant time and sex interaction effects on TpTe, TpTe/QT, TpTe/QTc, and TpTe/QRS (*P* < 0.01) and intergroup effects on TpTe, TpTe/QT, and TpTe/QTc (*P* < 0.05) (Table 2).

The results of further simple effect analysis showed that the time effect with time as an independent variable showed that the TpTe, TpTe/QT, TpTe/QTc, and TpTe/QRS of women at time 1, time 2, time 3 and time 4 were greater than those at time 0 (all *P* < 0.05), but there was no significant difference in male data at these five time points. The analysis of the intervention effect with group as the independent variable revealed significant differences in TpTe, TpTe/QT, TpTe/QTc, and TpTe/QRS between males and females at baseline (all *P* < 0 05). There was also a significant difference in TpTe/QRS between the sexes at time 1, but there was no significant difference between the sexes at any of the following time points (Table 2).

**Table 2:** Comparisons of electrocardiogram values and chlorpromazine equivalent dose between the two sexes at different timepoints

	Male	Female	F_time_	*P*_time_	F_sex_	*P*_sex_	F_time×sex_	*P*_time×sex_
Chlorpromazine equivalent dose(mg/d)			1.38	0.25	1.87	0.17	1.55	0.19
Baseline (time0) Time1 Time2 Time3 Time4	770.41 ± 321.77 713.27 ± 344.87 694.90 ± 317.58 660.20 ± 334.63 633.67 ± 319.41	716.03 ± 342.93 789.29 ± 412.62 800.00 ± 394.88 830.36 ± 381.84 714.29 ± 412.73						
QRS duration(ms)			1.38	0.24	22.16	0.00**	0.83	0.51
Baseline (time0) Time1 Time2 Time3 Time4	96.61 ± 8.88 96.08 ± 8.39 95.71 ± 9.65 93.90 ± 8.61 93.82 ± 7.56	87.54 ± 7.86 87.29 ± 9.09 87.68 ± 9.59 86.11 ± 9.40 87.82 ± 11.01						
RR interval(s)			1.92	0.11	0.48	0.49	1.72	0.16
Baseline (time0) Time1 Time2 Time3 Time4	0.72 ± 0.14 0.77 ± 0.14 0.79 ± 0.14 0.78 ± 0.16 0.74 ± 0.17	0.79 ± 0.20 0.76 ± 0.16 0.78 ± 0.14 0.80 ± 0.17 0.77 ± 0.17						
QT interval(ms)			3.95	0.00**	4.41	0.04*	0.21	0.93
Baseline (time0) Time1 Time2 Time3 Time4	360.80 ± 36.39 375.41 ± 35.84 379.98 ± 32.92 380.51 ± 34.37 379.82 ± 29.56	375.04 ± 49.42 384.96 ± 33.42 393.71 ± 50.12 392.86 ± 38.69 383.50 ± 33.86						
QTc interval(ms)			2.93	0.03*	2.41	0.12	1.39	0.23
Baseline (time0) Time1 Time2 Time3 Time4	427.69 ± 26.60 429.86 ± 29.75 430.88 ± 38.87 433.92 ± 37.61 445.29 ± 37.26	426.21 ± 28.84 446.61 ± 42.76 447.36 ± 47.26 440.96 ± 38.53 443.07 ± 41.95						
TpTe(ms)			9.71	0.00**	2.82	0.09	6.21	0.00**
Baseline (time0) Time1 Time2 Time3 Time4	117.18 ± 24.58a 116.71 ± 19.13 125.96 ± 28.07 125.35 ± 28.76 126.82 ± 29.14	85.39 ± 20.85a 123.79 ± 33.59b 115.29 ± 27.51b 117.18 ± 36.11b 121.79 ± 41.31b						
TpTe/QRS			11.22	0.00**	0.24	0.62	5.75	0.00**
Baseline (time0) Time1 Time2 Time3 Time4	1.22 ± 0.26a 1.22 ± 0.23a 1.34 ± 0.37 1.34 ± 0.31 1.36 ± 0.35	0.97 ± 0.21a 1.43 ± 0.38ab 1.32 ± 0.31b 1.37 ± 0.43b 1.41 ± 0.52b						
TpTe/QT			6.57	0.00**	7.82	0.01**	9.98	0.00**
Baseline (time0) Time1 Time2 Time3 Time4	0.33 ± 0.07a 0.31 ± 0.05 0.33 ± 0.06 0.33 ± 0.06 0.33 ± 0.07	0.23 ± 0.06a 0.32 ± 0.08b 0.29 ± 0.07b 0.30 ± 0.09b 0.32 ± 0.09b						
TpTe/QTc			9.10	0.00**	6.07	0.01**	6.17	0.00**
Baseline (time0) Time1 Time2 Time3 Time4	0.27 ± 0.05a 0.27 ± 0.04 0.29 ± 0.05 0.29 ± 0.06 0.29 ± 0.06	0.20 ± 0.05a 0.28 ± 0.06b 0.26 ± 0.06b 0.27 ± 0.08b 0.27 ± 0.09b						
iCEB			5.81	0.00**	25.93	0.00**	0.67	0.60
Baseline (time0) Time1 Time2 Time3 Time4	3.75 ± 0.52 3.93 ± 0.58 4.02 ± 0.58 4.09 ± 0.56 4.07 ± 0.45	4.30 ± 0.58 4.44 ± 0.49 4.52 ± 0.69 4.61 ± 0.59 4.42 ± 0.59						
iCEBc			3.62	0.01**	30.01	0.00**	1.12	0.34
Baseline (time0) Time1 Time2 Time3 Time4	4.44 ± 0.50 4.51 ± 0.51 4.55 ± 0.65 4.66 ± 0.56 4.77 ± 0.53	4.89 ± 0.46 5.15 ± 0.61 5.14 ± 0.64 5.17 ± 0.62 5.10 ± 0.61						

iCEB, index of cardiac electrophysiological balance; iCEBc, corrected iCEB; QTc, corrected QT interval.

Baseline (time 0), the data measured at admission; Time 1, one-year follow-up; Time 2, two-years follow-up; Time 3, three-years follow-up; Time 4, four-years follow-up.

*: *p* < 0.05; **: *p* < 0.01;

a: Comparison between groups, *p* < 0.05;

b: Comparison with baseline, *p* < 0.05.

### Effect of sex and time on ECG indices

**Fig. 3:**
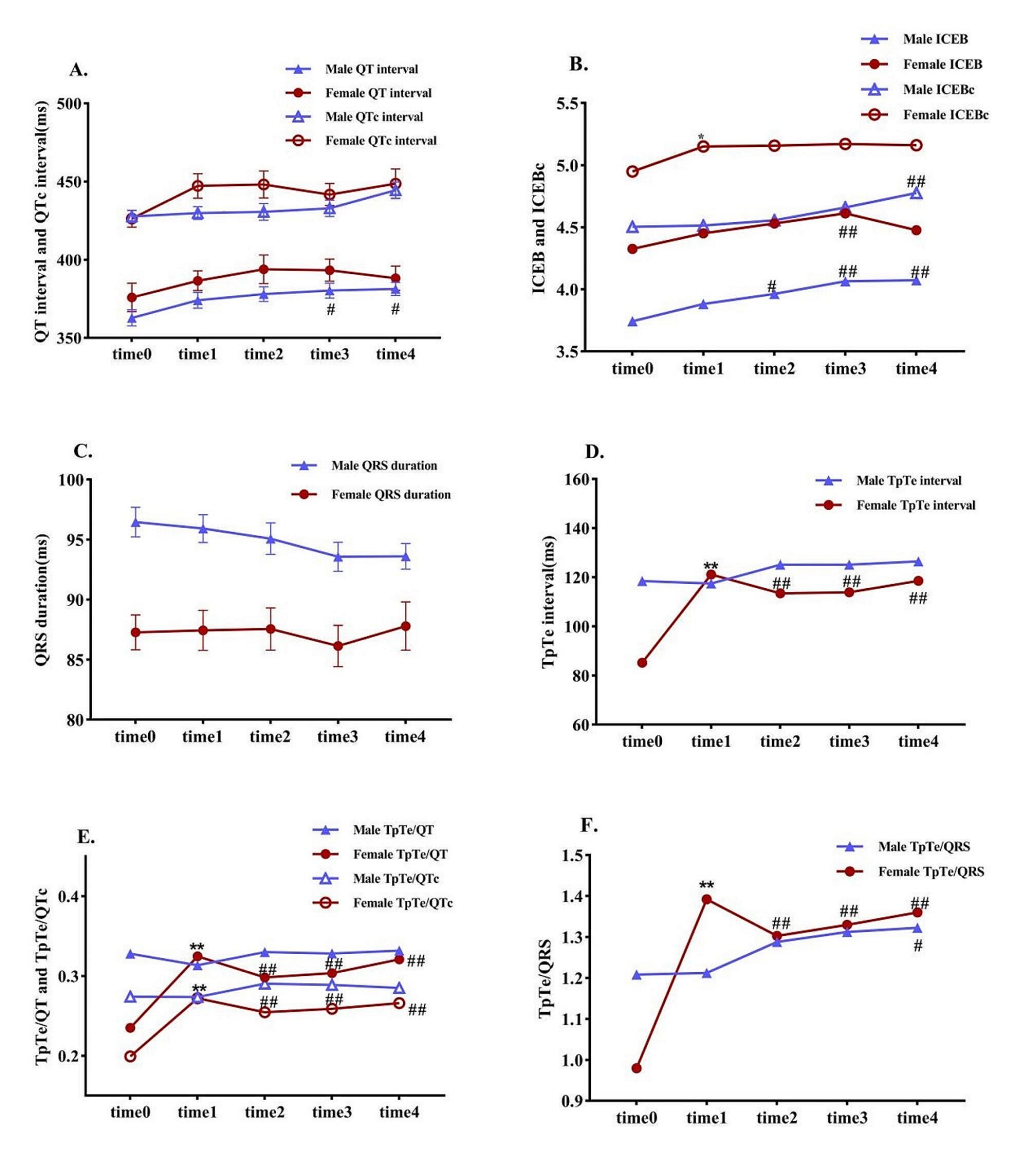
Comparison of ECG data between sexes. time 0, the data measured at admission; time 1, one-year follow-up; time 2, two-years follow-up; time 3, three-years follow-up; time 4, four-years follow-up. ** Compared with the previous time node, *P* < 0.01. ## Compared with baseline, *P* < 0.01. # Compared with baseline, *P* < 0.05

Men had a significantly longer QRS duration than women did (*P* < 0.01), but the QTc interval was similar between the sexes. At time 3, the male QT interval significantly differed from that at time 0, whereas the QT interval did not significantly change in female patients. Both iCEB and iCEBc changed significantly at time 3, but the pattern differed between sexes: male iCEB values at time 2 and time 3 were greater than those at time 0, especially at time 3 (*P* < 0.01). The iCEB of the females at time 3 was also significantly different from that at time 0 (*P* < 0.01). The male iCEBc was significantly greater at time 4 than at time 0 (*P* < 0.01), and the female iCEBc was significantly greater at time 1 than at time 0 (*P* < 0.05). There was a significant difference in the QRS duration between the two sexes (*P* < 0.01), but there was no significant difference between the different time points within either sex. The TpTe, TpTe/QT, TpTe/QRS and TpTe/QTc of women at time 1 were significantly greater than those at time 0 (*P* < 0.01), but these measures did not significantly change in men (Fig. 3).

### ROC curve of ventricular arrhythmia predicted by ECG indices of the two sexes

**Fig. 4:**
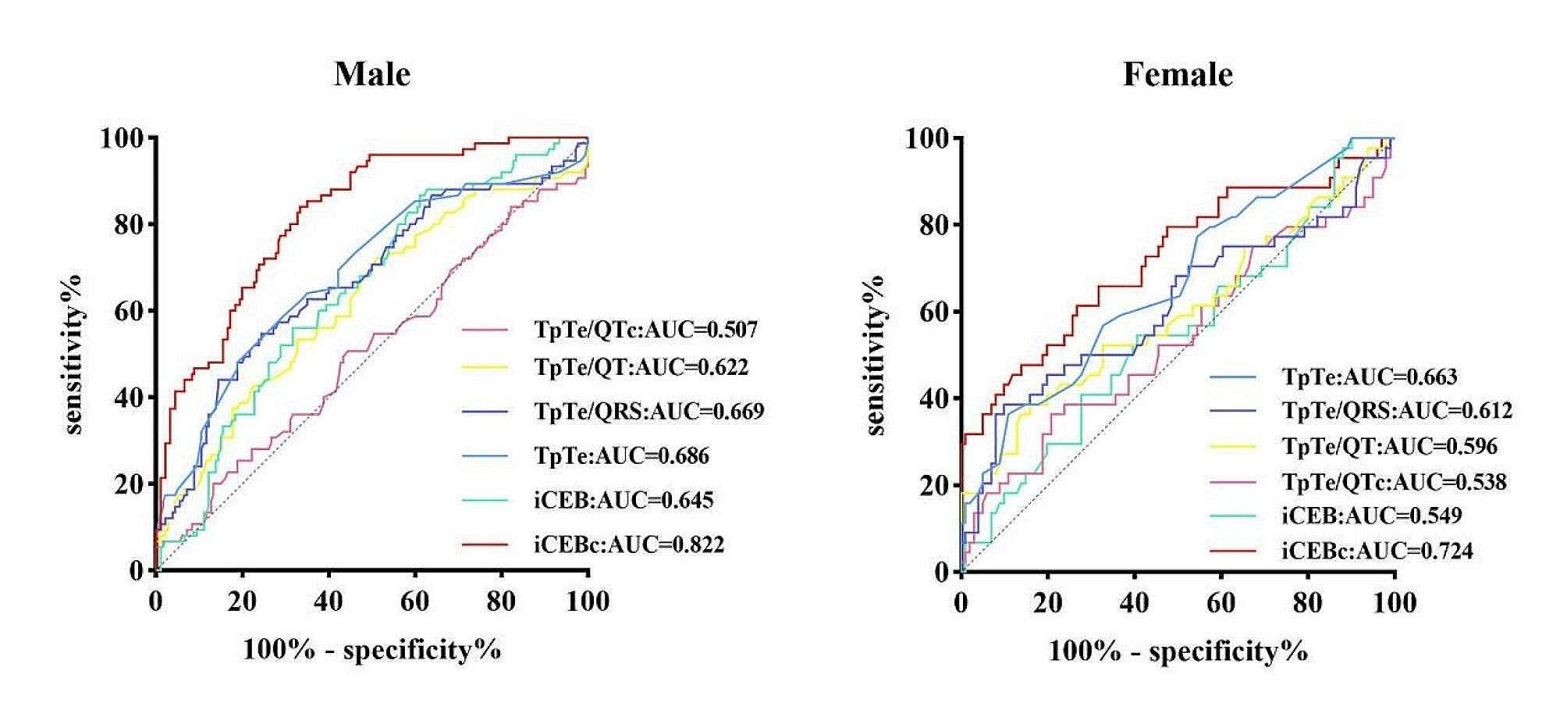
ROC curve of ventricular arrhythmia predicted by ECG indices in schizophrenia patients of different sexes

In schizophrenic patients, a QTc ≥ 450 ms in males and a QTc ≥ 470 ms in females were defined as malignant arrhythmias and were used as the cut-off points for generating the ROC curves (Fig. 4). According to the Youden index, the area under the curve (AUC) of male iCEBc was 82.2% (*P* < 0.001), and the best critical value for identifying malignant arrhythmias was 4.528, for which the sensitivity was 84% and the specificity was 67%. When the AUC of the female iCEBc was 72.4% (*P* < 0.001), the best critical value, sensitivity, and specificity for identifying malignant arrhythmias were 5.315%, 74% and 73%, respectively (Fig. 4).

### The incidence rate of cardiac arrhythmia

With prolonged treatment, the ventricular repolarization index increased, but no severe arrhythmia was found. The incidences of arrhythmia at 5 time points were 2.5%, 6.25%, 6.25%, 6.25% and 5% (arrhythmic events included premature ventricular beats and premature atrial beats). More than 90% of patients treated with antipsychotics did not have any arrhythmias.

## Discussion

Our study showed that iCEB and iCEBc changed before QT and QTc did. When there was a change in QTc, the difference between iCEB and iCEBc was more significant. The duration of ventricular repolarization gradually increased with the continuous use of antipsychotics, but there was no serious arrhythmia or sudden cardiac death during the 4-year observation period. The data suggest that antipsychotics are safe for a relatively long period.

The excitation conduction velocity and the effective refractory period of ventricular muscle excitation in the myocardium determine the propagation distance of excitation in the myocardium, that is, the wavelength of the heart (λ). The risk of TdP is usually detected by the use of drugs that increase heart wavelengths; drugs that reduce heart wavelengths are more likely to increase the risk of malignant ventricular arrhythmias in the occurrence of polymorphic ventricular tachycardia [19]. The use of cardiac wavelength as an indicator of risk stratification requires invasive detection methods. As a noninvasive and easily measured ECG parameter, a noninvasive and effective surrogate indicator of cardiac wavelength, the iCEB can reflect the imbalance between cardiac polarization and repolarization [20–22]. As a potential risk predictor of arrhythmia, the iCEB grew significantly longer during the 4-year observation period of this study, so we need to be vigilant. When the QRS time limit is constant, the QTc extension is greater than the QT extension, which increases the value of iCEBc and the slope of increase in the QTc extension compared with that of the iCEB, suggesting that the early warning value of iCEBc for arrhythmia may be better than that of iCEB.

The iCEBc was calculated based on the ratio of QTc to QRS (QTc/QRS). Our research showed that during the study period, the QTc increased with time but not significantly. The duration of QRS decreased with time, but there was no significant difference between sexes or at any of the time points. However, the changes in the iCEB and iCEBc were more significant, suggesting that the iCEBc is more sensitive than the QTc and has greater clinical value in predicting the risk of arrhythmias caused by antipsychotics. A study showed that iCEB and iCEBc increased after haemodialysis, but there was no significant change in the QT interval or QTc, which is consistent with our findings [23]. Some authors have explained differences in iCEBc by differences in QTc or QRS duration [24, 25]. With the application of antipsychotics, the QT interval prolongation, representing the total time of ventricular depolarization and repolarization, the QRS duration of ventricular polarization was shortened, and the iCEB (QT/QRS) and iCEBc (QT/QCRc) were significantly prolonged, indicating that the QT interval prolongation was caused by repolarization and that TpTe prolongation was the main factor leading to repolarization. If we focused only on the QT interval and ignored the imbalance between depolarization and repolarization, patients at high risk of malignant ventricular arrhythmia may be missed. However, the iCEBc may have greater clinical value in predicting antipsychotic drug risk. In this way, we predicted abnormal values of iCEBc. According to the abnormal QTc values in men and women, we predicted that the abnormal value of the iCEBc in males would be 4.528 and that in females would be 5.315.

Antipsychotics can prolong cardiac action potentials by blocking ion channels on the ventricular myocyte membrane, including sodium inwards current (I_Na_), L-type calcium current (I_Ca−L_) and a variety of potassium outwards currents. Among them, the rapidly activated delayed rectifier potassium current (I_Kr_) is most often inhibited by antipsychotics [26]. M cells are cardiomyocytes located in the middle layer of the ventricular wall and are the key to the development of drug-induced LQTS. Compared with those of endocardial and epicardial cells, the outwards potassium currents of M cells in stages 2 and 3 of repolarization were weaker, which led to a more obvious inhibitory effect of antipsychotics on the I_Kr_ of M cells [17]. Epicardial and endocardial repolarization end at the summit of the T wave, when M cells in the ventricular muscle layer begin to repolarize, and the time to the end of M cell repolarization is the TpTe interval [27, 28]. If the TpTe interval is prolonged, the degree of transmembrane diffusion of the action potential of ventricular myocytes increases, which may induce LQTS [29, 30]. The normal range of the TpTe interval is 80–100 ms, and a TpTe ≥ 103.3 ± 17.4 ms is considered to indicate longer follow-up [31]. In this study, the minimum and maximum average TpTe interval values in males (117.18 ± 24.58 ~ 126.82 ± 29.14 ms) exceeded the upper limit, as did those in females (115.29 ± 27.51 ~ 123.79 ± 33.59 ms). The TpTe interval of males gradually increased, while that of females significantly increased at the one-year follow-up and remained high afterwards, implying that the TpTe interval, an indicator of local ventricular myocardial repolarization, differed between men and women and that changes in the TpTe interval were more pronounced in women than in men during antipsychotic drug use.

Epicardial and endocardial cells showed less repolarization potential delay than M cells did, and the repolarization rate of M-cell action potentials continuously decreased in both males and females during the continuous use of antipsychotic drugs. These changes were even more pronounced in women, possibly because of the different effects of sex hormones on myocardial tissue. Androgen deprivation and anti-androgen therapy can prolong the TpTe interphase, while testosterone can shorten this interphase [32]. Some studies have shown that a decrease in progesterone in premenopausal women is related to prolongation of the T wave duration. Most of the women in this study were premenopausal, so the significant prolongation of TpTe may be related to the decrease in oestrogen [33]. TpTe, TpTe/QRS, TpTe/QT, and TpTe/QTc were significantly prolonged in females, and the extent of TpTe extension became more pronounced with drug treatment.

The patients in this study were hospitalized during the observation period, so their lifestyles were basically the same; for example, their mealtime, nutritional status, exercise time and bedtime were almost the same and were limited by ward management. The participants were prohibited from smoking or drinking alcohol, which controlled for the influence of confounding factors to some extent. In this study, a lower incidence of arrhythmias and a lower risk of malignant arrhythmias were observed. Young adults who were in good basic cardiac condition, who were maintaining electrolyte balance and who were maintaining effective and safe blood concentrations may help to reduce the occurrence of arrhythmias. During the study period, cardiac repolarization indices changed in these patients, and after excluding the influence of glycolipid metabolism factors, the abnormal results of cardiac repolarization indices persisted; therefore, considering the continuous use of drugs and the gradual prolongation of the ventricular repolarization index, it was necessary to conduct long-term monitoring to clarify the safety of long-term use of antipsychotics.

## Limitations

In this study, although the lifestyles of these patients remained unified, metabolic syndrome caused by antipsychotics could not be avoided, metabolic syndrome increased the cardiac burden, and although the effects of glycolipid metabolism were excluded during the study period, a longer period of observation may be needed [34]. In addition, this was a real-world study, and we did not intervene in the treatment regimens of clinicians who were treated with a combination of atypical antipsychotics with different mechanisms of action during the 4-year hospital stay or who had a change in medication due to efficacy, side effects, etc., which could have had an impact on the results. A meta-analysis published in The Lancet by Huhn et al. in 2019 showed that the atypical antipsychotics amisulpride and risperidone affect the QT interval, so we still need to pay attention to the effects of different classes of antipsychotics on CMAPs [35]. The potential risk of arrhythmia associated with changes in indices of ventricular repolarization and the mechanism of the effect of sex on ventricular repolarization require in-depth analyses in larger samples of patients.

## Conclusions

After long-term use of antipsychotics, the ventricular repolarization index gradually increased with time. The new ventricular repolarization indices iCEB and iCEBc were more sensitive than the QTc at predicting arrhythmia.

### Electronic supplementary material

Below is the link to the electronic supplementary material.

Supplementary Material 1

## Data Availability

All data generated or analysed during this study are included in this published article.

## Abbreviations

ECG: electrocardiogram
TdP: torsades depointes
LQTS: long QT syndrome
iCEB: index of cardiac electrophysiological balance
iCEBc: corrected iCEB
QTc: corrected QT interval
TG: triglyceride
TC: total cholesterol
HDL-c: high-density lipoprotein cholesterol
LDL-c: low-density lipoprotein cholesterol
AUC: area under curve
