# Supplementary material for: Effect of long-term use of antipsychotics on the ventricular repolarization index
Source: BMC Psychiatry. 2024 Jul 16;24:505. doi: 10.1186/s12888-024-05947-1 (PMC11250928; doi:10.1186/s12888-024-05947-1)
Supplement: Supplementary file 1 — Supplementary Material 1 [file 12888_2024_5947_MOESM1_ESM.docx]

**Attached table.**

Comparison of the effects of different types of drugs on ventricular depolarization and repolarization at different timepoints

These patients were treated with atypical antipsychotics during the study period and with combined therapy in the acute phase of the disease and were kept on a lower dose of drugs than the treatment dose after remission to the stable phase. During more than 5 years of hospitalization, these patients were treated with olanzapine, risperidone, aripiprazole, ziprasidone, clozapine, amisulpride, quetiapine fumarate, and paliperidone extended-release tablets. The Lancet by Huhn et al. in 2019 showed that atypical antipsychotics amisulpride and risperidone affect the QT interval,It had also been suggested that ziprasidone had an effect on QT interval. Therefore, the use of these three drugs was categorised as a group with an effect on the QT interval, and the use of other atypical antipsychotics was categorised as a group with no effect on the QT interval, and the differences in the indices of ventricular repolarisation were observed at different time points. Because of the difference in the normal value range of ventricular repolarisation indexes between men and women, tables were made for men and women separately(Table a. and Table b.).

The depolarisation index QRS duration differed between the two groups of men in the second and fourth years(*P*<0.05), the repolarisation indexes TpTe interval, TpTe/QRS, TpTe/QT, TpTe/QTc differed significantly in the second year(*P*<0.01), and the rest of the indexes did not differ significantly between the two groups at different time points.There was no significant difference in the QRS duration of depolarisation indicators between the two groups of women at different time points, and there was a significant difference in the QT interval of repolarisation indicators in the first year and the third year(*P*<0.01), and iCEB from the first year to the fourth year(*P*<0.05). there was a difference in the QTc interval in the third year(*P*<0.05), and the difference between the two groups of iCEBc was even more significant(*P*<0.01). there was a difference in the RR interval in the first year and the fourth year(*P*<0.05), and there were no significant differences in the rest of the indicators at different time points between the two groups. The effects of different drug classes on ventricular repolarisation indexes differed between men and women, with drugs affecting the QT interval having a greater effect on ventricular repolarisation indexes in women and a lesser effect in men possibly due to the protective effect of sex hormones.

Table a. Comparison of the effects of different types of drugs on ventricular depolarization and repolarization at different timepoints in men

|  | First year | | | | Second year | | | | Third year | | | | Fourth year | | | | Fifth year | | | |
| --- | --- | --- | --- | --- | --- | --- | --- | --- | --- | --- | --- | --- | --- | --- | --- | --- | --- | --- | --- | --- |
|  | Effect on the QT intervalMean(SD) | No effect on the QT intervalMean(SD) | *Z*/*t* | *p* | Effect on the QT intervalMean(SD) | No effect on the QT intervalMean(SD) | *Z/t* | *p* | Effect on the QT intervalMean(SD) | No effect on the QT intervalMean(SD) | *Z/t* | *p* | Effect on the QT intervalMean(SD) | No effect on the QT intervalMean(SD) | *Z/t* | *p* | Effect on the QT intervalMean(SD) | No effect on the QT intervalMean(SD) | *Z/t* | *p* |
| QRS duration(ms) | 94.14(7.89) | 96.82(8.81) | -0.74 | 0.71 | 94.50(7.14) | 96.04(8.42) | -2.10 | 0.04* | 89.60(10.07) | 96.13(9.38) | -1.47 | 0.15 | 90.67(6.74) | 93.96(8.81) | -2.16 | 0.03* | 92.53(7.28) | 94.06(7.78) | -0.65 | 0.52 |
| RR interval(s) | 0.69(0.20) | 0.72(0.13) | -0.47 | 0.64 | 0.77(0.12) | 0.77(0.14) | -0.05 | 0.96 | 0.77(0.15) | 0.78(0.15) | -0.18 | 0.86 | 0.73(0.11) | 0.79(0.16) | -0.80 | 0.43 | 0.71(0.13) | 0.77(0.18) | -1.07 | 0.29 |
| QT interval(ms) | 369.29(52.16) | 361.80(34.83) | 0.49 | 0.63 | 377.00(28.21) | 373.77(36.31) | 0.17 | 0.86 | 390.00(40.16) | 376.72(33.25) | 0.83 | 0.41 | 385.00(29.50) | 379.62(34.85) | 0.36 | 0.72 | 377.67(21.12) | 382.81(38.85) | -0.55 | 0.58 |
| QTc interval(ms) | 450.00(55.10) | 429.61(31.07) | 1.44 | 0.16 | 433.00(30.99) | 429.55(29.37) | 0.22 | 0.82 | 446.00(23.28) | 428.87(39.26) | 0.95 | 0.35 | 444.17(46.89) | 429.87(35.37) | 1.59 | 0.11 | 450.13(27.29) | 442.08(40.15) | 0.71 | 0.48 |
| TpTe(ms) | 125.00(31.89) | 117.43(24.32) | 0.73 | 0.47 | 142.00(19.65) | 115.34(18.20) | 2.79 | 0.01** | 140.00(52.32) | 123.52(24.35) | 1.26 | 0.21 | 117.50(21.15) | 126.16(29.06) | -0.70 | 0.49 | 124.53(27.25) | 127.28(29.48) | -0.31 | 0.76 |
| TpTe/QRS | 1.34(0.37) | 1.22(0.25) | 1.14 | 0.26 | 1.51(0.22) | 1.21(0.22) | 2.56 | 0.01** | 1.61(0.74) | 1.30(0.30) | 1.83 | 0.07 | 1.31(0.31) | 1.35(0.31) | -0.26 | 0.79 | 1.36(0.35) | 1.36(0.34) | -0.02 | 0.99 |
| TpTe/QT | 0.34(0.06) | 0.33(0.07) | 0.37 | 0.71 | 0.38(0.06) | 0.31(0.05) | 2.78 | 0.01** | 0.36(0.11) | 0.33(0.05) | 0.99 | 0.33 | 0.30(0.04) | 0.33(0.06) | -1.04 | 0.07 | 0.33(0.08) | 0.33(0.07) | -0.10 | 0.92 |
| TpTe/QTc | 0.28(0.05) | 0.27(0.05) | 0.08 | 0.94 | 0.33(0.03) | 0.27(0.04) | 2.71 | 0.01** | 0.31(0.10) | 0.29(0.05) | 0.90 | 0.37 | 0.26(0.04) | 0.29(0.06) | -1.39 | 0.17 | 0.28(0.06) | 0.29(0.06) | -0.63 | 0.53 |
| iCEB | 3.98(0.87) | 3.74(0.46) | 1.10 | 0.28 | 4.01(0.47) | 3.91(0.58) | 0.32 | 0.75 | 4.42(0.82) | 3.96(0.53) | 1.73 | 0.09 | 4.28(0.61) | 4.08(0.55) | 0.84 | 0.40 | 4.10(0.42) | 4.10(0.49) | 0.03 | 0.98 |
| iCEBc | 4.82(0.71) | 4.45(0.54) | 1.59 | 0.12 | 4.61(0.56) | 4.52(0.49) | 0.39 | 0.69 | 5.04(0.74) | 4.50(0.61) | 1.85 | 0.07 | 5.06(0.74) | 4.61(0.51) | 1.91 | 0.06 | 4.89(0.43) | 4.73(0.56) | 0.99 | 0.33 |
| Chlorpromazine equivalent dose(mg/d) | 735.71(359.07) | 784.09(314.34) | -0.37 | 0.71 | 500.00(355.90) | 741.49(377.08) | -1.37 | 0.17 | 730.00(670.45) | 704.35(268.29) | 0.17 | 0.87 | 525.00(267.82) | 685.56(335.18) | -1.12 | 0.27 | 553.33(285.65) | 670.83(325.00) | -1.22 | 0.23 |

iCEB, index of cardiac electrophysiological balance;iCEBc,corrected iCEB;QTc,corrected QT interval.

*:*p*<0.05*;***:*p*<0.01*.*

Table b.Comparison of the effects of different types of drugs on ventricular depolarization and repolarization at different timepoints in women

|  | First year | | | | Second year | | | | Third year | | | | Fourth year | | | | Fifth year | | | |
| --- | --- | --- | --- | --- | --- | --- | --- | --- | --- | --- | --- | --- | --- | --- | --- | --- | --- | --- | --- | --- |
|  | Effect on the QT intervalMean(SD) | No effect on the QT intervalMean(SD) | *Z*/*t* | *p* | Effect on the QT intervalMean(SD) | No effect on the QT intervalMean(SD) | *Z/t* | *p* | Effect on the QT intervalMean(SD) | No effect on the QT intervalMean(SD) | *Z/t* | *p* | Effect on the QT intervalMean(SD) | No effect on the QT intervalMean(SD) | *Z/t* | *p* | Effect on the QT intervalMean(SD) | No effect on the QT intervalMean(SD) | *Z/t* | *p* |
| QRS duration(ms) | 87.43(4.96) | 87.23(8.66) | 0.06 | 0.95 | 82.67(3.93) | 88.70(9.55) | -1.50 | 0.15 | 83.00(7.04) | 88.74(9.75) | -1.35 | 0.19 | 83.33(5.47) | 87.40(10.37) | -1.10 | 0.28 | 86.70(11.21) | 88.37(10.86) | -0.39 | 0.70 |
| RR interval(s) | 0.98(0.20) | 0.73(0.16) | 3.44 | 0.01** | 0.83(0.22) | 0.74(0.14) | 1.21 | 0.24 | 0.84(0.17) | 0.76(0.13) | 1.24 | 0.23 | 0.91(0.19) | 0.75(0.13) | 2.53 | 0.02* | 0.79(0.14) | 0.75(0.19) | 0.60 | 0.55 |
| QT interval(ms) | 420.00(47.26) | 361.86(40.93) | 3.16 | 0.01** | 396.67(53.07) | 383.87(27.99) | 0.82 | 0.42 | 442.00(30.74) | 381.39(45.55) | 3.06 | 0.01** | 407.67(43.89) | 386.80(34.36) | 1.39 | 0.18 | 386.70(26.32) | 389.00(48.69) | -0.14 | 0.89 |
| QTc interval(ms) | 432.41(20.75) | 427.91(35.44) | 0.34 | 0.74 | 437.17(25.19) | 449.83(45.59) | -0.65 | 0.52 | 488.33(64.11) | 437.57(35.61) | 2.61 | 0.02* | 432.44(44.00) | 445.85(35.48) | -0.87 | 0.39 | 437.80(35.78) | 454.32(57.35) | -0.83 | 0.42 |
| TpTe(ms) | 95.71(26.37) | 85.50(24.28) | 0.95 | 0.35 | 130.17(40.20) | 123.91(32.92) | 0.39 | 0.69 | 122.17(27.93) | 115.22(28.46) | 0.53 | 0.59 | 131.11(50.17) | 113.10(28.00) | 1.25 | 0.22 | 110.50(31.57) | 133.95(51.09) | -1.32 | 0.20 |
| TpTe/QRS | 1.09(0.26) | 0.98(0.29) | 0.85 | 0.41 | 1.58(0.49) | 1.40(0.35) | 1.01 | 0.32 | 1.48(0.37) | 1.30(0.30) | 1.24 | 0.23 | 1.56(0.55) | 1.31(0.36) | 1.49 | 0.15 | 1.31(0.46) | 1.54(0.61) | -1.04 | 0.31 |
| TpTe/QT | 0.23(0.06) | 0.24(0.07) | -0.34 | 0.74 | 0.33(0.08) | 0.32(0.08) | 0.09 | 0.93 | 0.28(0.06) | 0.30(0.07) | -0.87 | 0.39 | 0.32(0.11) | 0.30(0.08) | 0.68 | 0.50 | 0.29(0.08) | 0.34(0.10) | -1.45 | 0.16 |
| TpTe/QTc | 0.22(0.06) | 0.20(0.05) | 0.99 | 0.33 | 0.30(0.08) | 0.28(0.07) | 0.62 | 0.54 | 0.25(0.06) | 0.26(0.06) | -0.38 | 0.71 | 0.30(0.09) | 0.26(0.07) | 1.39 | 0.18 | 0.25(0.07) | 0.29(0.09) | -1.12 | 0.27 |
| iCEB | 4.80(0.39) | 4.18(0.57) | 2.70 | 0.01** | 4.81(0.66) | 4.36(0.41) | 2.10 | 0.04* | 5.29(0.41) | 4.33(0.59) | 3.74 | 0.00** | 4.94(0.61) | 4.47(0.52) | 2.15 | 0.04* | 4.51(0.51) | 4.46(0.73) | 0.19 | 0.85 |
| iCEBc | 4.96(0.35) | 4.94(0.58) | 0.08 | 0.94 | 5.29(0.38) | 5.11(0.64) | 0.67 | 0.51 | 5.89(0.59) | 4.97(0.50) | 3.86 | 0.00** | 5.19(0.74) | 5.16(0.66) | 0.17 | 0.87 | 5.10(0.54) | 5.19(0.77) | -0.36 | 0.72 |
| Chlorpromazine equivalent dose(mg/d) | 492.86(389.90) | 827.27(339.72) | -2.19 | 0.04* | 541.67(428.27) | 884.78.(404.92) | -1.83 | 0.08 | 775.00(348.93) | 823.91(412.55) | -0.27 | 0.79 | 638.89(293.45) | 945.00(396.00) | -2.07 | 0.05 | 570.00(294.58) | 826.32(461.99) | -1.59 | 0.12 |

iCEB, index of cardiac electrophysiological balance;iCEBc,corrected iCEB;QTc,corrected QT interval.

*:*p*<0.05*;***:*p*<0.01*.*
